# Risk factors for sub–therapeutic serum concentrations of magnesium sulfate in severe preeclampsia of Chinese patients

**DOI:** 10.1186/s12884-020-03277-0

**Published:** 2020-10-01

**Authors:** Jingjing Li, Lian Tang, Ruiheng Tang, Lan Peng, Liqiang Chai, Liping Zhu, Yanxia Yu

**Affiliations:** 1 Department of Pharmacy, The Affiliated Suzhou Hospital of Nanjing Medical University, Suzhou Municipal Hospital, Suzhou, 215002 Jiangsu China; 2 School of Medicine, Xuzhou Medical University, Xuzhou, 221000 Jiangsu China; 3 Department of Obstetrics, The Affiliated Suzhou Hospital of Nanjing Medical University, Suzhou Municipal Hospital, Suzhou, 215002 Jiangsu China

**Keywords:** Severe preeclampsia, Magnesium sulfate, Risk factors, Receiver operating characteristic curve, Logistic regression

## Abstract

**Background:**

Magnesium sulfate (MgSO_4_) is the standard drug for eclampsia prophylaxis and treatment. In China, the effective therapeutic serum magnesium level is 1.8–3.0 mmol/L. There is little information on how to achieve and maintain effective therapeutic concentrations. This study aimed to investigate risk factors for sub-therapeutic serum concentrations of MgSO_4_ in patients with severe preeclampsia.

**Methods:**

Patients with severe preeclampsia who received MgSO_4_ intravenous infusion were retrospectively reviewed. The maternal demographic characteristics, regimens for the administration of MgSO_4_, and lab test results of patients were collected. Multivariate logistic regression analysis and receiver operating characteristic (ROC) curve analysis were conducted for the risk factors influencing the serum magnesium concentration.

**Results:**

A total of 93 patients with severe preeclampsia were included in the study. 52 (55.91%) patients did not attain therapeutic serum magnesium levels. A multivariate logistic regression analysis identified creatinine clearance (Ccr), whether the loading dose was given, and measurement time of serum magnesium concentration (referring to the time from start of MgSO4 infusion to blood draw for serum sampling) as independent risk factors for sub**-**therapeutic serum magnesium concentration (*P <* 0.05). ROC curve analysis indicated that the continuous variable Ccr had a significant predictive value for the serum magnesium concentration, which resulted in a cutoff point of 133 mL/min; while measurement time had limited predictive value, with cutoff point of 2.375 h.

**Conclusions:**

Ccr, whether the loading dose was given, and measurement time were independent risk factors for sub**-**therapeutic serum magnesium concentration. A loading dose of MgSO_4_ everytime before the maintenance dose, as well as the duration of MgSO4 maintenance dose of more than 2.375 h are recommended for all the patients with severe PE. Routine evaluation of serum magnesium levels is a recommended practice for women with severe PE and whose Ccr is ≥133 mL/min.

## Background

Preeclampsia (PE) is a multi-system disorder of widespread vascular endothelial malfunction and vasospasm, characterized by elevation of blood pressure after 20 weeks of gestation in a formerly normotensive woman, with proteinuria, or in the absence of proteinuria, new-onset hypertension with the new onset of end-organ dysfunction, affecting 6–8% of all pregnancies [1]. Adverse outcomes tend to occur more frequently in severe cases of PE and eclampsia [2].

Magnesium sulfate (MgSO_4_) is the preferred pharmacological intervention to treat severe PE because it can prevent the recurrent seizures of eclampsia [2, 3]. Total dose of MgSO_4_ reported worldwide for the treatment of PE and eclampsia ranged from 2 g/24 h to 54 g/24 h [4–8]. Serum magnesium level of 2.0–3.5 mmol/L is considered therapeutic by several authors [8–14]. While in China, optimal control of convulsions is thought to be most effective with therapeutic serum magnesium level at 1.8–3.0 mmol/L. [4] Sub-therapeutic serum magnesium level may increase risk for eclamptic seizures [9]. On the other hand, MgSO_4_ overdose may result in serious toxicities, including maternal loss of the patellar reflex, respiratory paralysis, cardiac conduction and cardiac arrest [10, 15]. To date, there is little information on whether serum magnesium level can reach the effective therapeutic concentration and the influencing factors in patients with severe PE in China. In the present study, the clinical data of 93 patients with severe PE treated with MgSO_4_ were analyzed retrospectively to explore the risk factors for serum magnesium not reaching the therapeutic concentration.

## Methods

### Study population

The ethics committee of Suzhou Municipal Hospital approved our study protocol (K2017037). This was a retrospective analysis of electronic medical records of all women with severe PE admitted to our institution for delivery who received IV MgSO_4_ for seizure prophylaxis from January 2016 to December 2018. Verbal informed consent was obtained from all participants by telephone. Inclusion criteria were: (1) singleton pregnancy, (2) aged 18–45 years, (3) the baseline serum magnesium concentrations were measured before IV infusion of MgSO_4_, and (4) serum magnesium levels were measured during IV infusion of a maintenance dose. We excluded patients with multifetal pregnancies or other pregnancy complications, such as hepatic diseases, kidney diseases, etc. Diagnostic criteria of PE include the development of hypertension after 20 weeks of gestation in women with previously normal blood pressure, and proteinuria or in the absence of proteinuria, new-onset hypertension with new onset of thrombocytopenia, renal insufficiency, impaired liver function, pulmonary edema, cerebral or visual symptoms. The diagnostic standards of hypertension shall be in accordance with a systolic blood pressure of 140 mmHg or higher, or a diastolic blood pressure of 90 mmHg or higher on two occasions at least 4 h apart. Proteinuria is defined as the excretion of 0.3 g or more of protein in a 24-h urine collection. Alternatively, a protein/creatinine ratio of at least 0.3 (each measured as mg/dL) or dipstick test reading of 1+ is used. PE is diagnosed as severe based on classic criteria of blood pressures greater than or equal to 160/110 mmHg and proteinuria greater than or equal to dipstick reading of 2+. Other notable parameters symptoms are: persistent headache, visual disturbances, epigastric pain, intrauterine growth retardation and impaired hepatic and renal function tests [3, 4].

The patients with serum magnesium level 1.8 to 3.0 mmol/L after IV infusion of MgSO_4_ were assigned to Standard group, and those with serum magnesium level < 1.8 mmol/L were assigned to Sub-standard group. We collected data on maternal age, gestational age, height and weight, creatinine clearance (Ccr), alanine aminotransferase (ALT), aspartate aminotransferase (AST), albumin, baseline serum magnesium concentrations, whether the loading dose was given, and measurement time (referring to the time from start of MgSO_4_ infusion to blood draw for serum sampling.). The Ccr was calculated from the serum creatinine by the Cockcroft-Gault equation: $\mathrm{Ccr}=\frac{\left(140-\mathrm{Age}\right)\times \mathrm{body}\ \mathrm{weight}\left[\mathrm{kg}\right]}{\mathrm{Cr}\left[\mathrm{mg}/\mathrm{dL}\right]\times 72}$, where Cr was the serum creatinine. For women, the formula requires multiplication by 0.85.

### IV administration of MgSO_4_

A 5 g IV loading dose was or was not administered over 30 min, followed by a maintenance dose of 1.5 g/h for 10 h using an infusion pump (Terufusion infusion pump TE-135, Terumo Corporation, Tokyo, Japan).

### Measurement of magnesium level in the serum

Serum magnesium level was measured by 2 mL of venous blood sampling, which were collected into serum separator tubes (Becton Dickinson Franklin Lakes, NJ, USA). The blood samples were centrifuged at 3000 rpm for 5 min within 30 min of collection. Automatic biochemical analyzer (HITACHI 7600, Tokyo, Japan) was used to measure total magnesium, and serum magnesium concentrations of 1.8–3.0 mmol/L were considered therapeutic window for severe PE.

### Statistical analysis

On-admission factors, including age, height, weight, body mass index (BMI), gestational age, creatinine clearance (Ccr), alanine aminotransferase (ALT), aspartate aminotransferase (AST), albumin, baseline serum magnesium concentrations, measurement time, whether the loading dose was given, were expressed as number (%), mean ± standard deviation (SD) or median (quartile). Chi-square test was used in comparison of the parameter of whether the loading dose was given between the Standard group and Sub–standard group. For quantitative variables, the Kolmogorov-Smirnov test was used in the normality test. The age, weight, BMI, Ccr, ALT, AST, albumin, baseline serum magnesium concentrations, measurement time were not normally distributed, while the height and gestational age were with normal distribution. The Mann-Whitney U test was used to compare the parameters without normal distribution. The T-test was used in comparion of the data normally distributed between the Standard group and Sub–standard group. Univariate and multivariate analyses were performed to explore potential risk factors for sub-therapeutic blood magnesium concentration. Variables with a *P*-value < 0.1 on the univariate analysis were included in a multivariate logistic regression analysis. The weight, BMI, Ccr, albumin, measurement time and whether the loading dose was given were included into the multivariate logistic regression model to explore independent risk factors associated with sub-therapeutic blood magnesium concentration. We also calculated the odds ratio (OR) and 95% confidence intervals (CI). The area under ROC curve and the cut-off values were evaluated. Statistical analysis was proceeded using SPSS version 22.0 (RRID: SCR_002865). Differences with a *P*-value < 0.05 were considered statistically significant.

## Results

The study included ninety-three women with severe PE who received IV infusion of MgSO_4_ for seizure prophylaxis. Among these patients, there were 41 (44.09%) and 52 (55.91%) patients who did (Standard group) and did not (Sub**–**standard group) attain therapeutic serum magnesium levels. No one had the occurrence of seizure in the two groups studied during hospitalization. Table 1 shows the maternal demographic characteristics, lab test results, the regimens for the administration of MgSO_4_, and serum magnesium levels of the patients in the two groups. The Standard group and Sub**–**standard group showed no significant difference in age (28.00 vs. 31.00, *P* = 0.078), height (159.4 vs. 160.8, *P* = 0.109), BMI (27.79 vs.29.40, *P* = 0.090), gestational age (31.96 vs. 32.48, *P* = 0.285), ALT (28.00 vs. 24.50, *P* = 0.200), albumin (27.2 vs. 28.1, *P* = 0.084), and whether the loading dose was given (18 vs. 14, *P* = 0.087). The baseline serum magnesium concentrations were similar in both groups (0.76 vs. 0.73, *P* > 0.05). The median (quartile) serum magnesium concentration of women in Standard group was 2.08 (1.89, 2.25), while it was 1.39 (1.21, 1.61) for Sub**–**standard group. Women in Standard group had significantly lower weight (70.00 vs. 71.80, *P* = 0.048), lower Ccr (127 vs.162, *P* = 0.000), higher AST (30.00 vs. 26.50, *P* = 0.007), and higher measurement time (5.00 vs. 1.00, *P* = 0.013) than women in Sub**–**standard group (*P* < 0.05).

**Table 1:** Maternal demographic characteristics and serum magnesium levels of the patients in two groups

Variables	Standard group (***N*** = 41)	Sub–standard group (***N*** = 52)	***P***-value
Age (years)	28.00 (26.00, 33.50)	31.00 (28.25, 35.00)	0.078
Height (cm)	159.4 ± 4.4	160.8 ± 5.4	0.109
Weight (kg)	70.00 (65.00, 74.65)	71.80 (67.08, 84.50)	0.048
BMI (kg/m^2^)	27.79 (25.64, 29.43)	29.40 (25.67, 31.63)	0.090
Gestational age (weeks)	31.96 ± 3.65	32.48 ± 4.13	0.285
Ccr (mL/min)	127 (97, 155)	162 (132, 189)	0.000
ALT (U/L)	28.00 (21.50, 43.00)	24.50 (20.00, 37.50)	0.200
AST (U/L)	30.00 (22.00, 35.50)	26.50 (23.00, 36.50)	0.007
Albumin (g/L)	27.2 (24.6, 30.2)	28.1 (26.5, 31.8)	0.084
Baseline serum magnesium concentrations (mmol/L)	0.76 (0.71, 0.84)	0.73 (0.68, 0.81)	0.094
Measurement time (h)	5.00 (1.00, 7.00)	1.00 (0.50, 6.00)	0.013
Loading dose 5 g			0.087
Given (n)	18 (43.90%)	14 (26.92%)	
Not given (n)	23 (56.10%)	38 (73.08%)	

*BMI* Body mass index, *Ccr* Creatinine clearance, *ALT* Alanine aminotransferase, *AST* Aspartate aminotransferase

Through univariate analysis, we found that weight, BMI, Ccr, albumin, measurement time, and whether the loading dose was given were statistically significant risk factors for sub-therapeutic blood magnesium concentration (*P* < 0.1, Table 2). Multivariate regression analysis showed that Ccr (*P* = 0.000; 95% CI:1.008**–**1.030), whether the loading dose was given (*P* = 0.038; 95% CI:0.117**–**0.941) and measurement time (*P* = 0.008; 95% CI:0.688**–**0.947) were independent risk factors for sub-therapeutic blood magnesium concentration (Table 3).

**Table 2:** The results of univariate analysis for risk factors associated with sub-therapeutic blood magnesium concentration

Variables	OR	95%CI	***P***-value
Age	1.066	0.983–1.156	0.121
Height	1.059	0.973–1.152	0.184
Weight	1.047	1.006–1.090	0.024^*^
BMI	1.117	0.998–1.249	0.054^*^
Gestational age	1.035	0.931–1.150	0.524
Ccr	1.017	1.007–1.028	0.001^*^
ALT	0.989	0.973–1.005	0.176
AST	0.994	0.977–1.010	0.449
Albumin	1.099	0.995–1.214	0.062^*^
Maintenance dose	1.237	0.452–3.385	0.678
Measurement time	0.850	0.742–0.975	0.020^*^
Whether the loading dose was given	0.471	0.197–1.123	0.089^*^
Baseline serum magnesium concentrations	0.025	0.000–3.388	0.141

*OR* The odds ratio, *CI* Confidence intervals, *BMI* Body mass index, *Ccr* Creatinine clearance, *ALT* Alanine aminotransferase, *AST* Aspartate aminotransferase

^*^Variables with *P* value < 0.1

**Table 3:** Independent risk factors associated with sub-therapeutic blood magnesium concentration

Variables	OR	95% CI	***P***-value
Weight	/	/	0.467
BMI	/	/	0.774
Ccr	1.019	1.008 ~ 1.030	0.000^△^
Albumin	/	/	0.516
Measurement time	0.807	0.688 ~ 0.947	0.008^△^
Whether the loading dose was given	0.332	0.117 ~ 0.941	0.038^△^

*OR* The odds ratio, *CI* Confidence intervals, *BMI* Body mass index, *Ccr* Creatinine clearance

^∆^Statistically significant at *P* < 0.05

**Fig. 1:**
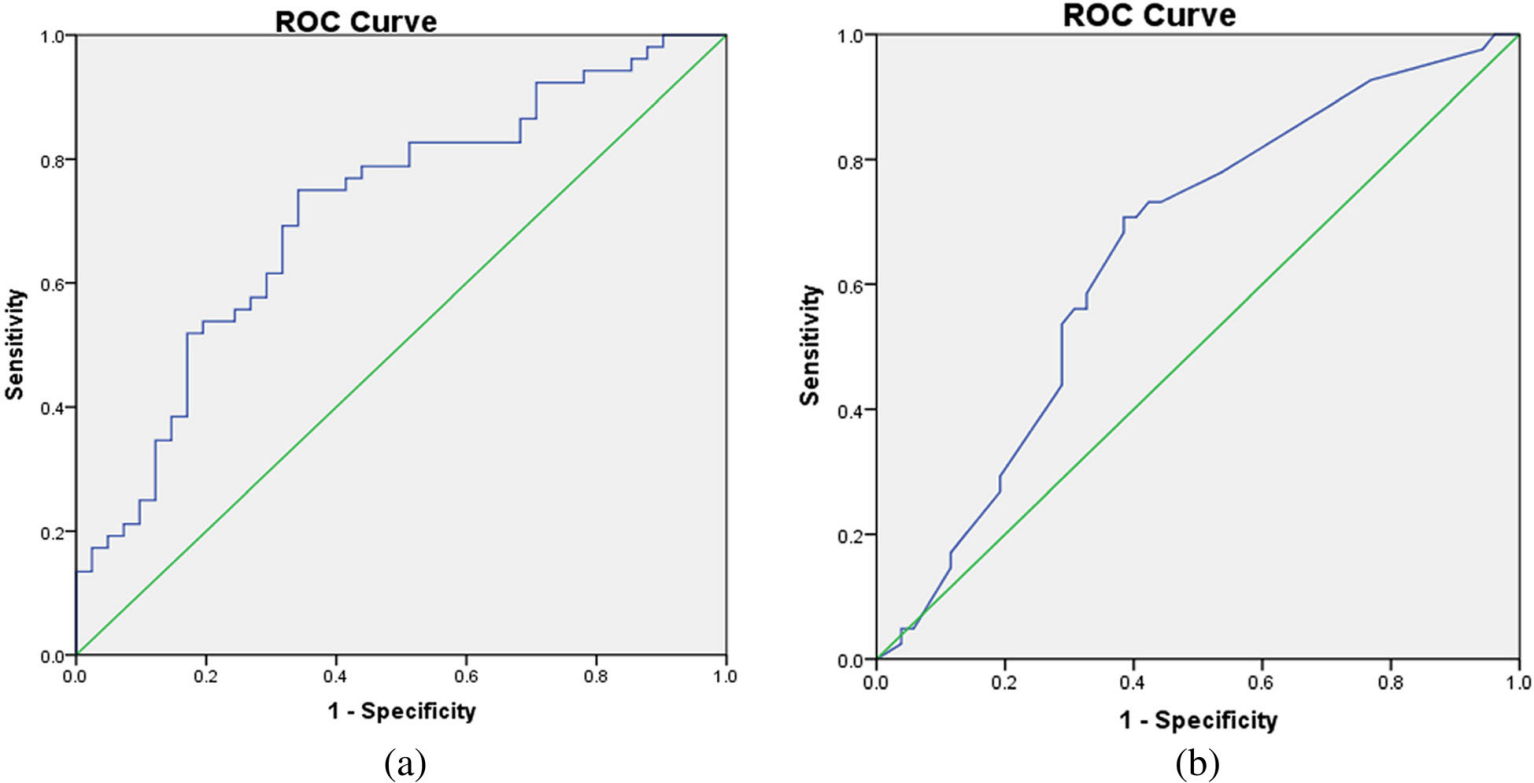
**a** The area under the ROC curve of Ccr was 0.715 with the cut-off value of 133 mL/min. **b** The area under the ROC curve of Measurement time was 0.650 with the cut-off value of 2.375 h

Independent risk factors of continuous variables were analyzed by ROC curve (Table 4). The area under ROC curve of Ccr was 0.715 with the cut-off value of 133 mL/min. The area under the ROC curve of measurement time was 0.650 with the cut-off value of 2.375 h (Fig. 1). The results showed that when Ccr ≥ 133 mL/min or the duration of MgSO_4_ maintenance dose was less than 2.375 h, the blood magnesium concentration was less likely to reach the target range of 1.8–3.0 mmol/L.

**Table 4:** Results of ROC curve analysis

Variables	Area under ROC curve	Youden index	Cut-off	Sensitivity/%	Specificity/%
Ccr	0.715	0.409	≥ 133(mL/min)	75.0	65.9
Measurement time	0.650	0.323	≤2.375(h)	70.7	61.5

*Ccr* Creatinine clearance, *ROC* Receiver operating characteristic

## Discussion

The results of this study indicated 52 (55.91%) patients did not attain therapeutic serum magnesium levels and maternal Ccr, whether the loading dose was given and measurement time were major determinants of attainment of therapeutic serum magnesium concentration.

The elimination of MgSO_4_ occurs primarily in the kidney, and PE associated renal damage can result in increased serum magnesium levels [16]. A previous publication showed that the glomerular filtration rate of normal pregnant women was 149 mL/min/1.73 m^2^ body surface area [17]. We used Ccr to estimate the glomerular filtration rate, which was calculated by the Cockcroft-Gault equation. From our study, the median (quartile) Ccr of Standard group was 127 (98, 155) mL/min, lower than normal pregnant women, while Sub–standard group was 162 (132, 189) mL/min (*P* < 0.05). This suggests the reverse association between Ccr and sub-therapeutic levels. Our study further found that when maternal Ccr ≥ 133 mL/min, the blood magnesium concentration of severe preeclampisa patients was less likely to reach the target range of 1.8–3.0 mmol/L. We prefer to recommend routine evaluation of serum magnesium levels in augmented renal clearance women because they are at significant risk for being sub-therapeutic. It is also necessary to observe closely for signs of toxicity in severe PE cases with delayed renal clearance of MgSO_4_.

The pharmacokinetic basis of MgSO_4_ dosing regimens for eclampsia prophylaxis and treatment is not clearly established [11], and there is no report of the time required to reach therapeutic range of serum magnesium concentration after the beginning of administration of maintenance dose in China. However, pharmacodynamics studies showed that with IV 4 g loading and 2 g/h maintenance dose, blood magnesium concentration was twice the baseline value within 30 min, and plateaued at 2–4 h with minimum fluctuation [5, 11, 18]. At 2 h after administration, serum magnesium ranged broadly from 1.0–3.5 mmol/L. [16] With our MgSO_4_ IV infusion regimen (5 g loading dose and 1.5 g/h maintained for 10 h, or no loading dose and 1.5 g/h maintained for 10 h), our data suggested that for the duration of MgSO_4_ maintenance dose of more than 2.375 h, the blood magnesium concentration was more likely to reach the target range of 1.8–3.0 mmol/L.

Our study is the first report on whether the serum magnesium during the maintenance administration of MgSO_4_ can reach the therapeutic range in patients with severe PE in China. Phuapradit and colleagues [19] reported that when the regimen of their patients with diagnosis of severe PE were given a 5 g MgSO_4_ intravenous bolus infusion and 1 g/h continous infusion and continued 24 h postpartum, only 56.2% patients had the serum magnesium concentration above the therapeutic level of 2.0–3.5 mmol/L. With our MgSO_4_ IV infusion regimen (5 g loading dose and 1.5 g/h maintained for 10 h, or no loading dose and 1.5 g/h maintained for 10 h), only 44.09% of patients attained therapeutic serum magnesium levels during IV infusion of a maintenance dose, which was similar with Phuapradit and colleagues^`^ report [19]. There is few patients attained therapeutic serum magnesium levels, the reason possibly related to lot of patients were not given the loading dose and MgSO_4_ is excreted by the kidneys. Whether the loading dose was given was confirmed to be one of the risk factors for sub**-**therapeutic serum magnesium concentration in our study. A total of 61 (65.59%) patients in the Standard group and Sub–standard group were not given the loading dose, which may be the major reason of low rate of patients attained therapeutic serum magnesium range. During pregnancy, kidney volume increases by up to 30% [20]. Renal plasma flow and glomerular filtration rate are also increased [21]. The excretion of MgSO_4_ may increase with the increase of glomerular filtration rate in the patients with severe PE.

It is generally believed that the baseline magnesium serum concentrations complicate the metabolism of MgSO_4_ [22]. The baseline serum concentrations may have influence on the serum magnesium concentration measured during IV infusion of a maintenance dose. The reported baseline serum magnesium concentrations were consistently < 1 mmol/L for women with PE and eclampsia [11]. Also, our study confirmed that the median (quartile) baseline serum magnesium concentration of women with severe PE in Standard group was 0.76 (0.71, 0.84), while 0.73 (0.68, 0.81) for Sub–standard group. The baseline serum magnesium concentration had no effect on the therapeutic serum magnesium concentration after administration of MgSO_4_ in our study. However, due to the small sample size of our study, the effect may have not been observed.

Previous studies reported disagreement as to the recommended IV administration dosage and therapeutic levels of MgSO_4_. Published dose regimens for MgSO_4_ vary widely, with loading doses of 4–6 g intravenously over 20–30 min and maintenance doses of 1–2 g/h (and up to 3 g/h) [8, 12]. The most common MgSO_4_ regimen is a loading dose of 6 g intravenously over 15 to 20 min followed by 2 g/h as a continuous infusion [2, 23–25]. A therapeutic range of 2.0–3.5 mmol/L has been recommended based on retrospective data [9]. However, Chinese guidelines for the diagnosis and treatment of hypertension and preeclampsia in pregnancy recommend the therapeutic serum magnesium level of 1.8–3.0 mmol/L, with a loading dose 2.5–5 g and a maintenance dose of 1–2 g/h for 6–12 h [4]. But the guideline does not clearly state that the loading dose needs to be given every day before the maintenance dose of MgSO_4_ is administered. Therefore, a loading dose of MgSO_4_ is usually only administered to the patients who begin to receive treatment of MgSO_4_ for eclampsia prophylaxis on the first day in Chinese clinical practice. Our study found that patients with severe PE who were not given a loading dose were less likely to reach the target serum magnesium range. It has been repeatedly shown that the protocol of 4 g loading and 2 g/h maintenance infusion in preeclampsia-eclampsia patients can attain better therapeutic levels of serum magnesium compared to other protocols with no detectable difference in maternal and neonatal outcomes [9, 26]. Hence, we may consider recommending that a loading dose be used before the maintenance dose of MgSO_4_ is administered every time in China.

The association between elevated BMI and sub-therapeutic MgSO_4_ levels was not confirmed in our research, which was inconsistent with previous reports [16, 27, 28]. The reason may be related to the small sample of cases, which lead to the weakening of statistical significance. In addition, maternal BMI is correlated with gestational age, so the effects of these two parameters on serum magnesium levels cannot be clearly differentiated. Our study found there was no significant difference between Standard group and Sub–standard group regarding gestational age, which may result in no significant impact of BMI on serum magnesium levels.

The major strengths of the present study are as following. First, this is the first report on whether the serum magnesium during the maintenance administration of MgSO4 reaching the therapeutic range in patients with severe PE in China. Second, the present results are useful to the clinical practice. A loading dose of MgSO_4_ is recommended to be administered everytime before the maintenance dose to achieve target serum magnesium concentration range of 1.8–3.0 mmol/L.

There are limitations to this retrospective study due to limited clinical data. (1) Its retrospective nature precluded the best assessment methodology. And because of the small sample size, we did not observe a significant difference in efficacy of seizure prevention between the two groups. (2) It was inevitable that some variables were absent because the existing data was collected from medical record retrospectively. Fortunately, most required information in this study was included in the medical records. (3) We excluded the severe PE patients with serious co-morbidities such as hepatic diseases, kidney diseases, etc. The exclusion of these patients may limit our data collection. (4) Including subjects who did not receive a loading dose may dilute findings from those who received a standard approach. Because of the small sample size, the ROC curves generated were not particularly strong for a predictive test. Further prospective cohort studies with a larger sample size are necessary to draw any definitive conclusions on these issues. (5) The minimum effective treatment concentration of MgSO_4_ for prophylaxis and treatment of severe PE has largely been based on clinical and laboratory observations in earlier studies rather than standard exposure-response studies [6, 12] .Although some pharmacokinetic studies of MgSO_4_ administration in preeclamptic women are reported [29–32], there has been no rigorous evaluation of therapeutic serum magnesium concentration [11]. In the future, we will conduct a prospective study on whether patients with severe PE can achieve effective treatment concentration with MgSO_4_. Due to the complexity of the use of MgSO_4_ in the world, we also want to further study the impact of MgSO_4_ IV infusion regimen with or without loading dose on the blood concentration.

## Conclusions

In conclusion, the incidence of sub-therapeutic serum magnesium concentration during the maintenance administration in Chinese severe PE patients is high and associated with Ccr, whether the loading dose was given, and measurement time. Thus, to achieve targeted therapeutic serum magnesium concentrations, we recommend a loading dose of MgSO_4_ everytime before, as well as the duration of MgSO_4_ maintenance dose of more than 2.375 h for all the patients with severe PE. Women with severe PE and whose Ccr is ≥133 mL/min are recommended to do routine evaluation of serum magnesium levels.

## Data Availability

The data of this study is available from the corresponding authors on reasonable request.

## Abbreviations

ALT: Alanine aminotransferase
AST: Aspartate aminotransferase
BMI: Body mass index
Ccr: Creatinine clearance
CI: Confidence intervals
IV: Intravenous
MgSO_4_: Magnesium sulfate
OR: Odds ratio
PE: Preeclampsia
ROC: Receiver operating characteristic
SD: Standard deviation
